# Salmonella Infection Arising From a Wound in an Infant: A Case Report

**DOI:** 10.7759/cureus.84044

**Published:** 2025-05-13

**Authors:** Abdullah F Alnofie, Jamal D Alrobaiee, Ahmed E Aljohani, Hussain R Barnawi

**Affiliations:** 1 Laboratory Medicine, Armed Forces Hospital, Taif, SAU

**Keywords:** bilateral hand cellulitis, case report, pediatric cellulitis, pediatric infectious diseases, rare salmonella presentations, salmonella, salmonella group d, soft tissue infections

## Abstract

Bilateral hand cellulitis is an uncommon manifestation of *Salmonella* infection, particularly in pediatric patients. While *Salmonella* is typically associated with gastrointestinal symptoms, extraintestinal infections such as cellulitis are rare and pose diagnostic challenges. These atypical presentations highlight the clinical diversity of *Salmonella* species and underscore the need for timely and targeted management.

A previously healthy one-year-old girl presented with progressive swelling, redness, and warmth in both hands, accompanied by fever. There was no history of trauma, insect bites, or gastrointestinal symptoms. Initial laboratory investigations revealed no systemic abnormalities, and wound cultures identified *Salmonella* Group D as the causative pathogen. The infection did not respond to initial empirical topical therapy, necessitating a transition to targeted systemic antibiotics based on culture sensitivity. Given the severity of the condition and poor response to conservative management, the patient underwent bilateral incision and drainage under general anesthesia. Intraoperative findings confirmed the diagnosis, and postoperative care included wound dressing, pain management, and supportive measures. The patient showed steady improvement, with complete resolution of symptoms following a full course of antibiotics and surgical intervention.

This case underscores the importance of considering *Salmonella* in the differential diagnosis of cellulitis, even in pediatric patients without typical risk factors such as trauma or immunosuppression. Early diagnosis, pathogen-directed antibiotic therapy, and surgical intervention are essential for managing such rare presentations. Increased awareness and parental education on infection prevention are critical to reducing the burden of *Salmonella*-related complications in vulnerable populations. This case contributes to the growing literature on the diverse clinical presentations of *Salmonella*.

## Introduction

*Salmonella* infections are a significant global health concern, affecting millions annually, with the highest burden seen in developing countries [1]. While gastrointestinal manifestations are the most common clinical presentation, *Salmonella* species, particularly non-typhoidal *Salmonella* (NTS), have demonstrated the ability to cause rare extraintestinal infections, including osteomyelitis, abscesses, and cellulitis [2]. These atypical presentations are often challenging to diagnose, especially in pediatric populations where immune systems are still maturing and clinical manifestations may overlap with other conditions.

Localized infections caused by NTS, such as *Salmonella* Group D, are typically associated with predisposing factors such as immunosuppression, trauma, or invasive medical devices [3]. However, cases occurring in otherwise healthy individuals highlight the pathogen’s clinical diversity and its capacity to invade beyond the gastrointestinal tract. Pediatric presentations of NTS-associated cellulitis are exceedingly rare, with few documented cases in the literature [4], emphasizing the need for heightened clinical suspicion and timely intervention.

The pathogenesis of NTS extraintestinal infections is believed to involve transient bacteremia or direct inoculation, often following environmental exposure. These infections may mimic common bacterial cellulitis caused by organisms such as *Staphylococcus aureus* or *Streptococcus pyogenes*, leading to potential delays in diagnosis and treatment [5].

This case report contributes to the growing body of knowledge on *Salmonella* infections by detailing a unique case of bilateral hand cellulitis caused by *Salmonella* Group D in an otherwise healthy one-year-old child. The report highlights the critical importance of comprehensive diagnostic evaluations, pathogen-specific therapy, and the role of surgical intervention in managing severe soft tissue infections.

## Case presentation

A previously healthy one-year-old girl presented to the ED with swelling and redness in her left hand, noted a day after visiting a garden. The parents reported no history of trauma, insect bites, or other known injuries. Her initial assessment included a physical examination and imaging. An X-ray showed no abnormalities, and the child was discharged home with paracetamol for symptomatic relief.

Two days later, the child returned to the ED with worsening symptoms, including bilateral hand swelling, redness, and warmth, accompanied by a fever of 39°C. She had decreased activity and poor oral intake, with parents describing her as "less responsive than usual." No other symptoms, such as cough, diarrhea, vomiting, or changes in urination, were reported. There were no gastrointestinal symptoms that would typically suggest a primary source of *Salmonella* infection. A diagnosis of bilateral hand cellulitis was made, and the child was admitted for further evaluation and treatment.

Investigations

Upon admission, initial laboratory tests showed significant leukocytosis, with a WBC count of 17.7 × 10⁹/L and an elevated neutrophil percentage of 59.1%. Mild anemia was observed, with a hemoglobin level of 9.3 g/dL, and the platelet count was 483 × 10⁹/L. C-reactive protein (CRP) was markedly elevated at 34.6 mg/L, indicating significant inflammation (Table 1).

**Table 1:** Patient’s laboratory report. NEU: Neutrophils; %N: Percentage of Neutrophils; LYM: Lymphocytes; %L: Percentage of Lymphocytes; MONO: Monocytes; %M: Percentage of Monocytes; EOS: Eosinophils; %E: Percentage of Eosinophils; BASO: Basophils; %B: Percentage of Basophils; HGB: Hemoglobin; HCT: Hematocrit; MCV: Mean Corpuscular Volume; MCH: Mean Corpuscular Hemoglobin; MCHC: Mean Corpuscular Hemoglobin Concentration; RDW: Red Cell Distribution Width; PLT: Platelets; MPV: Mean Platelet Volume; CRP: C-Reactive Protein.

Parameter	Value	Reference Range
WBC (×10⁹/L)	17.7	4.0-11.0
NEU (×10⁹/L)	10.49	1.5-7.7
% Neutrophils	59.1	40-80
LYM (×10⁹/L)	5.38	1.0-4.8
% Lymphocytes	30.4	20-45
MONO (×10⁹/L)	1.77	0.2-1.0
% Monocytes	9.99	2-10
EOS (×10⁹/L)	0.04	0.0-0.5
% Eosinophils	0.25	0.0-0.5
BASO (×10⁹/L)	0.06	0.0-0.5
% Basophils	0.34	0.0-1.0
RBC (×10¹²/L)	3.49	3.8-6.0
HGB (g/dL)	9.3	11.5-15.5
HCT (L/L)	0.28	0.36-0.50
MCV (fL)	79.5	80-96
MCH (pg)	26.8	27-34
MCHC (g/dL)	33.7	31-36
RDW (%)	19	11.5-14.5
PLT (×10⁹/L)	483	150-400
MPV (fL)	11.1	9.0-12.0
CRP (mg/L)	34.6	0.0-5.0

**Figure 1:**
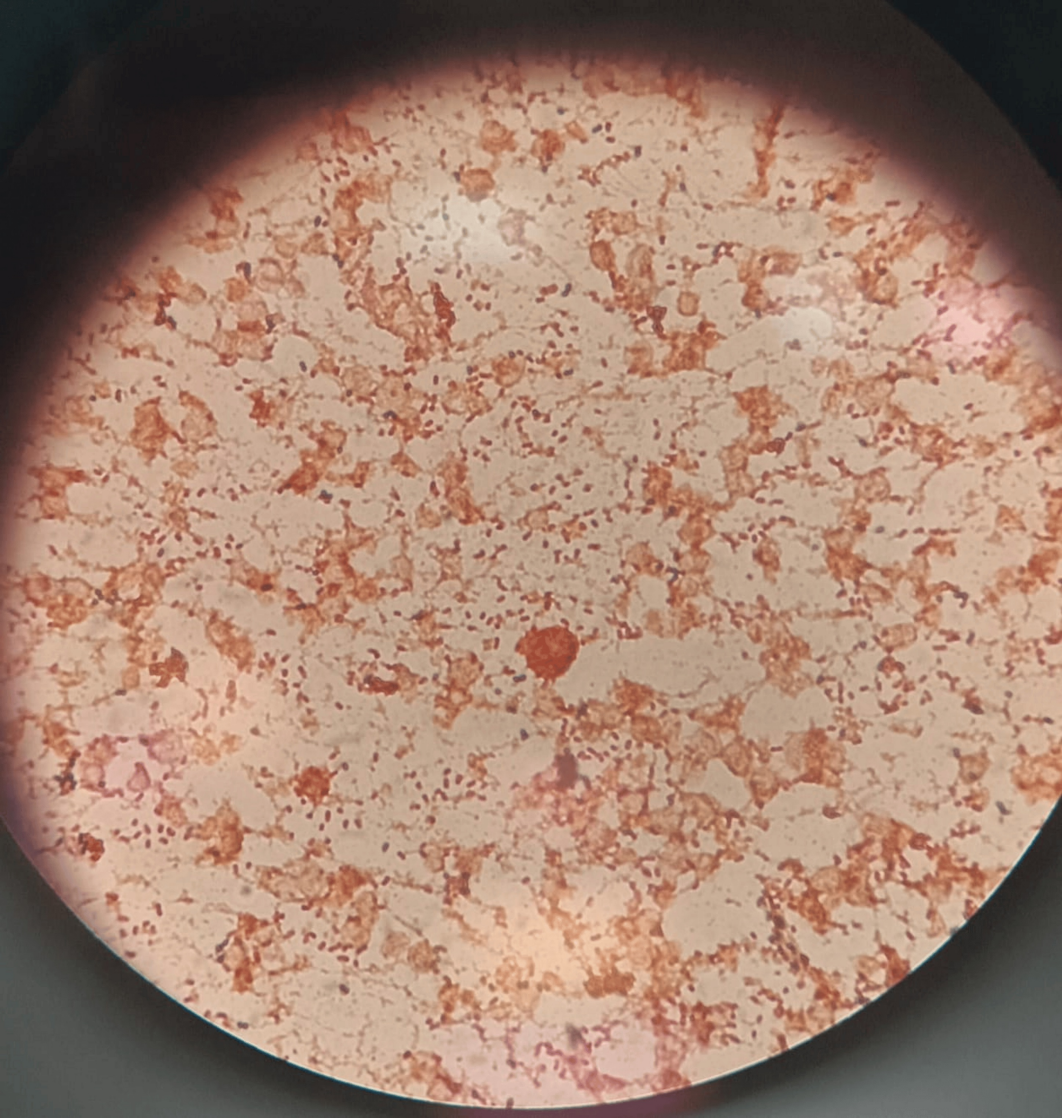
Gram stain of the wound specimen showing Gram-negative bacilli, consistent with Salmonella Group D.

**Figure 2:**
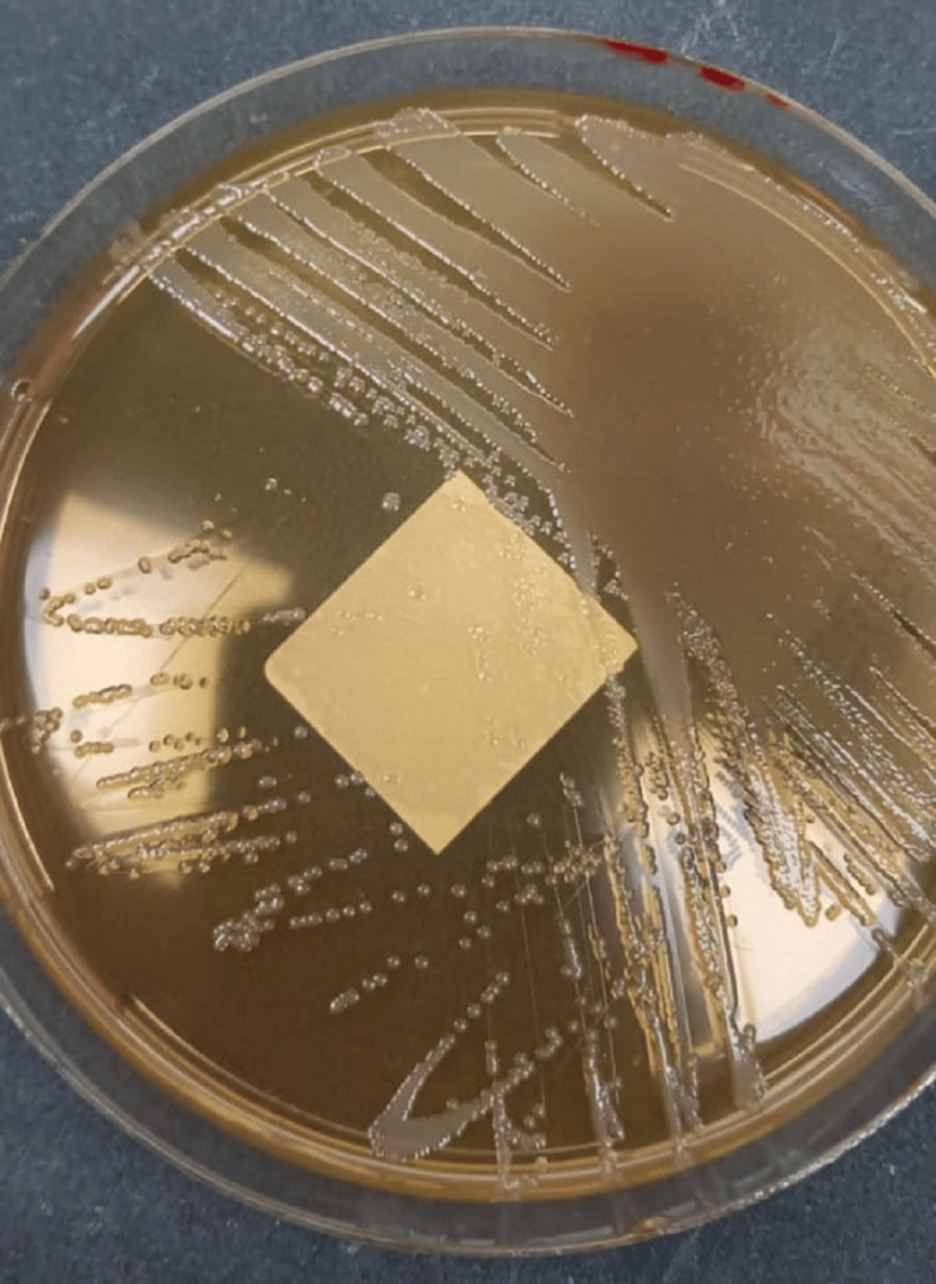
Colonies of Salmonella Group D on MacConkey agar. The colonies exhibit characteristic morphology, confirming bacterial growth from the wound swab culture.

Blood cultures and pus from the hand swelling were collected for microbiological analysis. Biochemical testing and VITEK analysis of the wound culture confirmed the presence of *Salmonella* Group D (Figures 1-2). Blood cultures did not grow *Salmonella*, suggesting the infection was localized to the soft tissues of the hands. Stool cultures were negative, and no diarrhea or gastrointestinal symptoms were reported, ruling out gastrointestinal seeding as a primary source of infection.

A complete metabolic panel revealed no major abnormalities aside from mild hypoalbuminemia (albumin: 38 g/L) and elevated bilirubin (total bilirubin: 15.3 µmol/L), likely reflecting the inflammatory response.

Imaging studies ruled out bone involvement or abscess formation beyond the soft tissue, and the diagnosis of bilateral soft tissue cellulitis secondary to *Salmonella* Group D was confirmed.

Therapeutic intervention

The patient was initially started on empirical antibiotic therapy with topical fusidic acid cream and intravenous ceftriaxone while awaiting culture and sensitivity results. However, due to the rapid progression of symptoms over the course of two days and the severity of the cellulitis, empirical treatment was escalated to systemic broad-spectrum antibiotics with coverage for potential Gram-negative pathogens.

Once the wound culture results confirmed *Salmonella* Group D, the antibiotic regimen was tailored to include intravenous ceftriaxone, a third-generation cephalosporin with proven efficacy against *Salmonella*. The dosing was optimized for her weight and age to ensure therapeutic levels. Collaboration with an infectious disease specialist ensured the regimen was effective while minimizing the risk of resistance.

Supportive measures, including intravenous fluids to maintain hydration and reduce inflammation, were also administered. Pain management was provided using acetaminophen and ibuprofen, titrated to her symptoms.

During her hospital stay, the patient showed steady improvement. Swelling and redness of both hands gradually subsided, and her fever resolved within 48 hours of surgical intervention. Repeat blood work showed a decrease in inflammatory markers, including normalization of CRP.

The patient completed a 10-day course of intravenous ceftriaxone, followed by an additional 7-day course of oral antibiotics to ensure eradication of the infection. Wound healing progressed well, with no signs of residual infection, scarring, or functional impairment of the hands.

Given the severity of the infection and the lack of significant improvement with medical therapy alone, the decision was made to proceed with surgical intervention. On the third hospital day, the patient underwent bilateral incision and drainage under general anesthesia.

Intraoperatively, copious purulent material was drained from both hands. Samples were sent for microbiological analysis, which reconfirmed *Salmonella* Group D. The tissues appeared inflamed but without necrosis or deeper abscesses involving muscles or bones. Postoperatively, the wounds were irrigated and dressed with antibiotic-impregnated gauze.

Post-surgical care included daily wound dressing changes, close monitoring for signs of reinfection or systemic spread, and continuation of systemic antibiotic therapy. Pain control was achieved with regular acetaminophen and nonsteroidal anti-inflammatory drugs (NSAIDs).

Parental education on hand hygiene and environmental infection prevention was provided prior to discharge. The parents were advised to avoid potential sources of contamination, such as improperly cooked food or unsanitary conditions during play.

At her two-week follow-up, the child showed complete resolution of symptoms, with no recurrence of swelling or redness. Follow-up laboratory tests, including WBC count and CRP, were within normal ranges. At the one-month follow-up, the patient continued to thrive, with no evidence of recurrence or complications.

## Discussion

NTS infections are predominantly known for their gastrointestinal manifestations, yet extraintestinal presentations such as soft tissue infections, including cellulitis, are rare. NTS species, such as *Salmonella* Group D, are associated with a broad clinical spectrum ranging from self-limiting gastroenteritis to invasive diseases like bacteremia, osteomyelitis, and soft tissue infections, including rare cases of thoracic spine osteomyelitis [6]. Soft tissue infections caused by NTS are exceedingly uncommon, accounting for approximately 1.5% of cases in a large cohort of patients with NTS infections.

This case highlights the clinical diversity of NTS infections by documenting bilateral hand cellulitis in a previously healthy one-year-old girl. The absence of risk factors such as immunosuppression, trauma, or predisposing comorbidities makes this presentation particularly unique. Most cases of *Salmonella*-associated soft tissue infections occur in immunocompromised individuals or following direct inoculation from penetrating injuries. In this patient, no clear source of infection was identified, although environmental exposure from a garden visit was considered a potential risk factor.

The pathogenesis of NTS soft tissue infections involves either hematogenous seeding during transient bacteremia or direct inoculation through a disrupted skin barrier. In children, the immature immune system may contribute to increased susceptibility to infections caused by NTS. This case underscores the importance of maintaining a broad differential diagnosis for pediatric cellulitis, particularly when initial empirical treatment fails or the clinical presentation is atypical.

A thorough diagnostic approach is critical in cases of atypical cellulitis. In this patient, the combination of clinical features, elevated inflammatory markers (e.g., CRP), and definitive identification of *Salmonella* Group D through wound culture enabled a precise diagnosis. The utility of obtaining wound cultures cannot be overstated, as they provide both confirmation of the causative agent and guidance for targeted antimicrobial therapy. Negative stool cultures in this case further emphasize the localized nature of the infection, which may occur even in the absence of systemic dissemination.

The treatment of NTS soft tissue infections involves two essential components: antimicrobial therapy and surgical management. Initial empirical antibiotic therapy must provide adequate coverage for Gram-negative bacteria, particularly *Salmonella*. In this case, the patient was started on intravenous ceftriaxone, which remains a first-line choice for invasive NTS infections due to its efficacy and favorable safety profile in pediatric patients. Targeted therapy based on culture sensitivity results ensures optimal outcomes and minimizes the risk of antibiotic resistance.

Surgical management is often necessary in severe soft tissue infections to remove purulent material, reduce bacterial load, and prevent complications. In this case, the bilateral incision and drainage procedure proved to be a critical component of the therapeutic approach, allowing for symptom resolution and facilitating wound healing. Surgical intervention is particularly important in pediatric patients with localized collections of pus or refractory cellulitis that does not respond to medical therapy alone.

The prognosis for *Salmonella*-induced soft tissue infections in immunocompetent pediatric patients is generally favorable with timely and appropriate management. However, delayed diagnosis or inadequate treatment can lead to serious complications, including systemic dissemination, abscess formation, or tissue necrosis. A similar case of postauricular extradural abscess caused by *Salmonella* in a toddler was reported by Lim CC et al., further highlighting the variability in clinical presentation and severity [7]. This case underscores the importance of early recognition, multidisciplinary management, and comprehensive follow-up in achieving successful outcomes.

## Conclusions

This case highlights the rare presentation of *Salmonella* Group D-induced bilateral hand cellulitis in an otherwise healthy pediatric patient. It underscores the importance of considering *Salmonella* as a potential pathogen in cases of cellulitis, even in the absence of gastrointestinal symptoms or identifiable risk factors. Comprehensive diagnostic evaluation, pathogen-specific antimicrobial therapy, and timely surgical intervention are paramount in ensuring favorable outcomes. Further studies are warranted to better understand the pathogenesis, risk factors, and optimal management of *Salmonella*-associated soft tissue infections in pediatric populations.
